# Galectin-9 is a restrictor of infection with multiple enteroviruses

**DOI:** 10.1128/jvi.00179-26

**Published:** 2026-03-27

**Authors:** Zichun Xiang, Guanying Wang, Wenjing Wang, Zhongqin Tian, Sunyuan Liu, Xia Xiao, Lili Ren, Zhuo Zhou, Xiaobo Lei, Jianwei Wang

**Affiliations:** 1 State Key Laboratory of Respiratory Health and Multimorbidity, and Christophe Médrieux Laboratory, National Institute of Pathogen Biology, Chinese Academy of Medical Sciences & Peking Union Medical College, Beijing, People's Republic of China; 2 NHC Key Laboratory of System Biology of Pathogens, Key Laboratory of Pathogen Infection Prevention and Control (Ministry of Education), National Institute of Pathogen Biology, Chinese Academy of Medical Sciences & Peking Union Medical College, Beijing, China; 3 Key Laboratory of Respiratory Disease Pathogenomics, Chinese Academy of Medical Sciences & Peking Union Medical College, Beijing, People's Republic of China; 4 Institute of Systems Medicine, Chinese Academy of Medical Sciences, Suzhou, Jiangsu, People's Republic of China; University of Michigan Medical School, Ann Arbor, Michigan, USA

**Keywords:** enteroviruses, galectin 9, VP2

## Abstract

Enteroviruses (EVs), encompassing over 200 sero/genotypes, cause diseases ranging from mild illness to severe systemic infections. Galectin-9 (Gal-9) is an immunomodulatory lectin implicated in various viral infections, yet its role in EVs remains unexplored. Here, we identify Gal-9 as a novel host restriction factor against a broad spectrum of EVs, including EV-A71, EV-D68, and Coxsackievirus B2. *LGALS9* deficiency increased viral replication in cells and pathogenicity in a mouse model of EV-A71 infection, while its overexpression inhibited viral replication. Mechanistically, Gal-9 specifically interacts with the viral VP2 protein, and mapping identified residues 179–194 of Gal-9 as critical for this interaction. A Gal-9 Δ179–194 mutant lost VP2-binding capacity and antiviral activity. These findings reveal that Gal-9 is a key component of the host antiviral response against EVs, functioning through a direct interaction with the viral VP2 protein.

**IMPORTANCE:**

Enteroviruses (EVs) cause a wide spectrum of disease and present a persistent global health challenge, highlighted by the cyclical resurgence of strains like EV-D68 and EV-A71. However, due to a lack of sufficient understanding of their pathogenesis, no EV-specific antiviral drugs are available. This study demonstrates that galectin-9 (Gal-9) is a key host factor that broadly antagonizes EVs, including EV-A71, EV-D68, and Coxsackievirus B2. We show that Gal-9 deficiency enhances viral replication both in cell culture and in an animal model. By identifying Gal-9 as a crucial restriction factor against EVs, our study provides foundational insight for the future development of antiviral strategies.

## INTRODUCTION

Enteroviruses (EVs), belonging to the *Picornaviridae* family, encompass numerous serotypes distributed across multiple species, including seven primary species affecting humans (*alphacoxsackie, betacoxsackie, coxsackiepol, deconjuncti, alpharhino, betarhino,* and *cerhino*). EVs are responsible for a wide range of diseases, from mild illnesses such as the common cold to severe conditions such as acute flaccid myelitis and poliomyelitis, contributing to substantial morbidity and economic burden. Poliovirus (PV), a member of the *Enterovirus coxsackiepol*, has been effectively controlled through widespread vaccine availability. However, in the post-polio era, acute flaccid myelitis has become strongly associated with EV-D68 and EV-A71 infections (1). In 2014, EV-D68 caused a nationwide outbreak in the United States, leading to severe respiratory illness and polio-like acute flaccid paralysis (2). Since then, EV-D68 has reappeared in a biennial cycle worldwide (3–7). EV-A71, another major pathogen, is a leading cause of hand, foot, and mouth disease (HFMD), a potentially life-threatening disease that predominantly affects infants and young children across the world (1, 8). Severe HFMD could lead to poor health-related quality of life and the economic burden (9). Therefore, EVs have a significant impact on global public health. Despite their considerable burden, there are currently no EV-specific antiviral drugs available, largely because of an insufficient understanding of EV pathogenesis.

The genome of EVs consists of a single positive-strand RNA molecule. This viral RNA encodes a single open reading frame that is translated into a large polyprotein. Through proteolytic cleavage, the polyprotein generates the structural proteins VP1, VP0, and VP3, along with the non-structural proteins 2A, 2B, 2C, 3A, 3B, 3C, and 3D. VP1, VP0, and VP3 self-assemble to form the capsid of the virion (10). During viral maturation, the precursor protein VP0 is autocleaved, yielding the mature structural proteins VP4 and VP2. VP1 and VP2 play critical roles in mediating virus-receptor interactions and initiating infection. Specific mutations of VP2_149M_ and VP1_145E_ in EV-A71 have been shown to cooperatively enhance viral binding and RNA accumulation (11). Non-structural proteins are involved in viral replication, transcription, and processing.

Galectins are a family of 16 carbohydrate-binding proteins characterized by one or two conserved carbohydrate recognition domains (CRDs), which are named numerically (12). The sequence identity among the CRDs of various galectins ranges from 20% to 50%, which results in distinct carbohydrate-binding preferences and diverse biological functions (13). Galectins are primarily localized in the cytoplasm but can be secreted via non-classical pathways and function extracellularly despite lacking a signal sequence (13). Studies have shown that galectins play diverse roles in regulating enteroviral infections. For instance, galectin-1 (Gal-1) facilitates EV-A71 infection by binding to the viral proteins VP1 and VP3 via its CRDs. This interaction enables Gal-1 to be released and subsequently bind to the surface of new target cells along with the virus (14). Similarly, Gal-3 has been shown to promote EV-A71 replication in a cellular model. Interestingly, the rs4644 AA genotype, a common variant of Gal-3, may exert a protective effect (15). Gal-9 has two CRDs connected by a linker domain. Gal-9 has six isoforms generated by alternative splicing: full, Δ5, Δ5/6, Δ5/10, Δ5/6/10, and Δ6. Their molecular weights range from 25 to 39 kDa (16). It is encoded by the *LGALS9*, which is ubiquitously expressed in various tissues and cells (17), especially in the lymphatic and digestive systems (18, 19). The overall sequence identity between mouse and human Gal-9 is approximately 70%. Importantly, the amino acid residues around the carbohydrate-binding site are highly conserved, indicating that both mouse Gal-9 and human Gal-9 likely recognize the same physiological targets and serve similar biological roles (18, 20, 21). Furthermore, the N-terminal CRD exhibits a higher carbohydrate-binding affinity than the C-terminal CRD (22). Gal-9 has many physiological functions including cell growth, differentiation, death, cell adhesion, and immunomodulation (17, 23). Gal-9 promotes inflammation by activating inflammatory cytokines in monocytes (24) and promoting maturation of dendritic cells and macrophages (25, 26). In addition, Gal-9 plays an anti-inflammatory role by inducing apoptosis of activated T cells (CD8^+^ and CD4^+^) (27) and promoting the differentiation of naïve T cells into regulatory T cells by amplifying Foxp3 expression (28). Gal-9 limits inflammation by promoting protein degradation of NLRP3 in primary peritoneal macrophages (29).

Several studies have investigated the role of Gal-9 in human viral infections. Gal-9 is upregulated in the trigeminal ganglion (TG) after HSV-1 infection. Gal-9 inhibits the antiviral function of TG-resident CD8^+^ T cells by binding to Tim-3-expressing TG-resident CD8^+^ T cells and inducing their apoptosis (30). Plasma Gal-9 concentrations are upregulated in response to human cytomegalovirus (HCMV) infection. Gal-9 inhibits HCMV by restricting viral fusion, which is dependent on the CRD (31). The concentrations of Gal-9 are increased in the sera of patients with active chronic hepatitis B virus (HBV) infection-related liver inflammation (32). In HBV-infected hepatocytes, Gal-9 (the sole ISG member of the galectin family) (33) and viperin, two type I IFN-induced expression proteins, act synergistically to direct the autophagic degradation of HBV core protein (HBc) to exert antiviral effects (34). Plasma levels of Gal-9 are elevated in COVID-19 patients, and this increase correlates positively with disease severity (35). Gal-9 potently enhances SARS-CoV-2 replication in human airway epithelial cells by promoting SARS-CoV-2 attachment and entry into AECs in an angiotensin-converting enzyme 2 (ACE2)-dependent manner (36).

To date, the role of Gal-9 in EV infection has not been demonstrated. To address this gap, we investigated the role of Gal-9 in EV-A71 replication using *LGALS9* knockout mice and cells. Our data showed that Gal-9 interacts with the VP2 proteins of EV-A71, EV-D68, and Coxsackievirus B2 (CV-B2) to restrict their replication.

## RESULTS

### Gal-9 exerted an inhibitory effect on EV-A71, EV-D68, and coxsackievirus B2 (CV-B2), but not on poliovirus 3 (PV3) in cells

**Fig 1:**
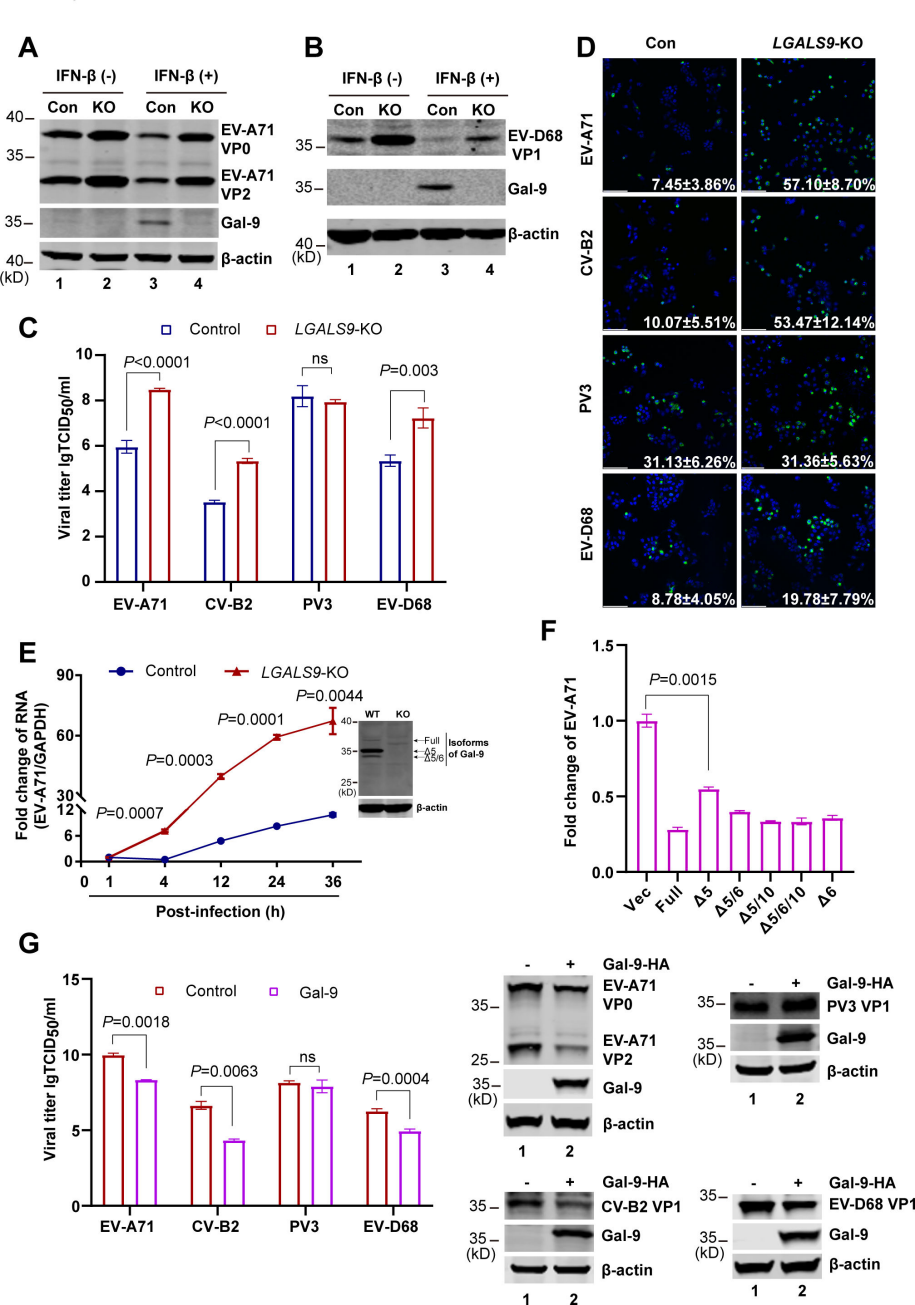
Gal-9 exerted an inhibitory effect on EV-A71, EV-D68, and coxsackievirus B2 (CV-B2), but not on poliovirus 3 (PV3) in cells. (**A**) *LGALS9*-KO and control A549 cells were stimulated with 50 U/mL IFN-β. After 24 h stimulation, cells were infected with 0.1 MOI EV-A71 for 12 h. Cell lysates were collected and subjected to WB with antibodies against VP0/VP2, Gal-9, and β-actin. (**B**) *LGALS9*-KO and control A549 cells were stimulated with 50 U/mL IFN-β. After 24 h stimulation, cells were infected with 0.5 MOI EV-D68 for 12 h. Cell lysates were subjected to WB with antibodies against VP1, Gal-9, and β-actin. (**C**) *LGALS9*-KO and control A549 cells were infected with EV-A71, CV-B2, PV3, and EV-D68 for 24 h. Supernatants were collected, and viral titers were detected by using the TCID_50_ assay. (**D**) *LGALS9*-KO and control A549 cells were infected with EV-A71, CV-B2, PV3, and EV-D68. At 24 h post-infection, the cells were immunostained with dsRNA (green) and DAPI. Cell images were scanned using a Leica TCS SP8. Eighteen fields were imaged to calculate the percentages in each well. Scale bar, 5 μm. (**E**) *LGALS9*-KO and control THP-1 cells, which were differentiated into macrophages using PMA, were infected with EV-A71 for the indicated times. Total RNA extracted from cells was evaluated by qRT-PCR using the SYBR Green method. The data are expressed as fold changes of the RNA levels of EVs relative to the *GAPDH* control. (**F**) 293T cells were transfected with vector, 6 isoforms of Gal-9 for 24 h. Cells were then infected with 0.01 MOI of EV-A71. At 24 h after infection, total RNA extracted from cells was evaluated by qRT-PCR using the SYBR Green method. (**G**) 293T cells were transfected with vector, isoform Δ5 of Gal-9-HA for 24 h. Cells were then infected with EV-A71, CV-B2, PV3, and EV-D68. At 24 h after infection, supernatants were collected to detect viral titers by using the TCID_50_ assay, and cell lysates were analyzed by WB using antibodies against HA, β-actin, and capsid protein of viruses.

We used a library of 146 ISG-knockout A549 cells to screen for host factors affecting EV-D68 replication and found that *LGALS9* knockout promoted EV-D68 replication. To further validate the role of *LGALS9* against enteroviruses, we infected control and *LGALS9*-KO A549 cells with EV-A71 and EV-D68 with or without IFN-β treatment. Western blot analysis showed that EV-A71 and EV-D68 replicated more efficiently in *LGALS9*-KO A549 cells than in the control cells (lanes 1 and 2 in Fig. 1A and B). IFN-β treatment induces the expression of hundreds of ISGs, including the Gal-9 (Δ5) isoform. Notably, this did not obscure the antiviral effect of LGALS9 against either EV-A71 or EV-D68 (lanes 3 and 4 in Fig. 1A and B), suggesting that *LGALS9* is the critical antiviral effector downstream of IFNs.

To determine whether *LGALS9* is a broad-spectrum restriction factor against EVs, we selected EV-A71, CV-B2, PV3, and EV-D68 as representatives of *Enterovirus alphacoxsackie, Enterovirus betacoxsackie, Enterovirus coxsackiepol,* and *Enterovirus deconjuncti*, respectively, to assess Gal-9’s antiviral activity. Twenty-four hours post-infection (hpi), titration experiments revealed that *LGALS9* deficiency significantly increased the replication of EV-A71, CV-B2, and EV-D68, but not that of PV3 (Fig. 1C). This finding was confirmed by immunofluorescence (Fig. 1D). Given that *LGALS9* is most abundantly expressed in monocytes, we generated *LGALS9* knockout THP-1 cell lines using CRISPR/Cas9 gene-editing technology. In wild-type THP-1 cells, the Δ5 isoform (about 35 kDa) was the predominantly expressed Gal-9 variant, whereas no expression was detected in the knockout cells (Fig. 1E). Consistent with our finding in epithelial cells, EV-A71 replication was significantly enhanced in *LGALS9*-KO THP-1 cells compared with WT controls (Fig. 1E).

Before assessing the antiviral effect of Gal-9 ectopic expression, we first sought to identify which isoform is capable of suppressing enteroviral replication. RD cells exogenously expressing the six Gal-9 isoforms were infected with EV-A71. Twenty-four hours post-infection (hpi), qRT-PCR analysis revealed that all isoforms of Gal-9 could inhibit the replication of EV-A71 (Fig. 1F). Since the Δ5 isoform was the predominantly expressed variant in our cellular models (Fig. 1A, B, and E), all subsequent overexpression and truncation experiments were conducted using this Δ5 isoform. Consistent with the results in *LGALS9*-KO cells, ectopic expression of Gal-9 inhibited the replication of EV-A71, CV-B2, and EV-D68 but had no effect on PV3 in viral titer assays (Fig. 1G). Taken together, our observations suggest that Gal-9 serves as a potent antiviral host factor against multiple EVs, including EV-D68, EV-A71, and CV-B2, but not PV3. The absence of Gal-9’s inhibitory effect on PV3 cannot be attributed to poor viral replication in A549 cells, as both poliovirus and other enteroviruses have been confirmed to propagate in this cell line (37).

### Gal-9 restricted EV-A71 RNA replication

**Fig 2:**
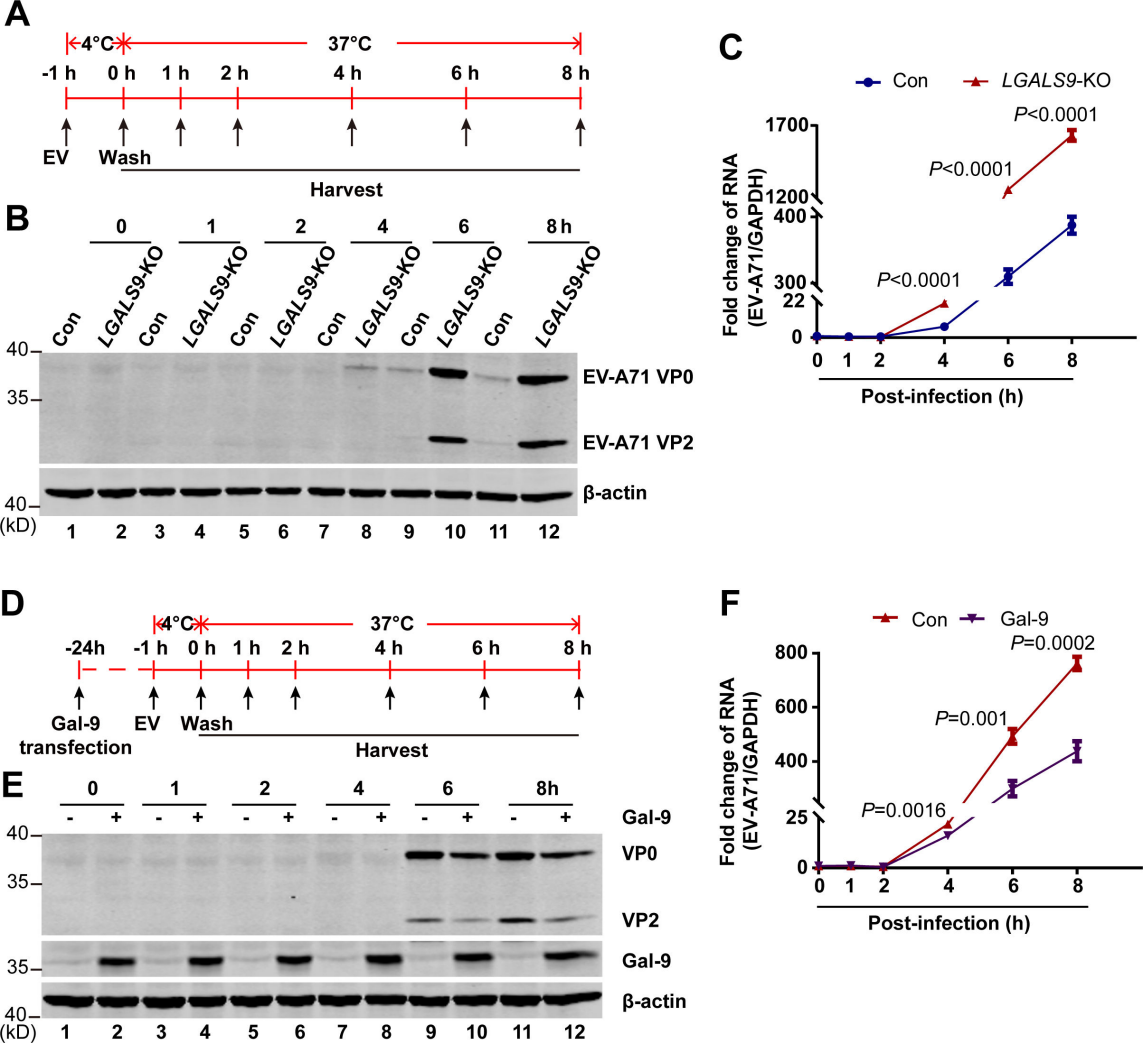
Gal-9 exhibits an inhibitory effect on viral replication at 4 h post-EV-A71 infection. (**A**) Schematic of EV-A71 infection in *LGALS9*-KO and control A549 cells. EV-A71 was allowed to bind to the cells on ice for 1 h. After washes, infection was allowed to proceed by incubating the cells at 37°C and collecting at indicated times. (**B and C**) *LGALS9*-KO and control A549 cells were infected with 0.5 MOI EV-A71 and performed as indicated in panel A. WB (**B**) and qRT-PCR (**C**) were performed. (**D**) Schematic of EV-A71 infection in *LGALS9*-KO A549 cells with Gal-9 transfection. *LGALS9*-KO A549 cells transfected with isoform Δ5 of Gal-9-Flag plasmids. After 24 h transfection, 0.5 MOI EV-A71 was allowed to bind to the cells on ice for 1 h. After washes, infection was allowed to proceed by incubating the cells at 37°C and collecting them at indicated times. (**E and F**) *LGALS9*-KO A549 cells were transfected with Gal-9 plasmids and infected with 0.5 MOI EV-A71 and performed as indicated in panel E. WB (**E**) and qRT-PCR (**F**) were performed. All experiments were done at least twice, and one representative is shown. Error bars indicate SD of technical triplicates. *P*-values of the two-tailed Student’s *t*-test are indicated.

To determine the stages of the viral life cycle that Gal-9 targets, we first examined early events prior to or after viral entry. As illustrated in Fig. 2A, EV-A71 infection was synchronized on ice for 1 h, followed by incubation at 37°C for the indicated time points. Western blot analysis showed that the structural protein VP0 was detectable in *LGALS9*-KO cells but not in wild-type cells at 4 hpi. At 6 and 8 hpi, KO cells had much higher levels of VP0 and VP2 proteins than wild-type cells (Fig. 2B). Next, we examined viral RNA levels in both cell types using qRT-PCR analysis. At 4 hpi, viral RNA levels were higher in KO cells than in wild-type cells (Fig. 2C). Consistently, as illustrated in Fig. 2D, re-expression of Gal-9 in *LGALS9*-KO cells reduced VP0 and VP2 protein levels at 6 h and reduced viral RNA accumulation at 4 h (Fig. 2E and F). These results suggest that Gal-9 inhibits EV infection after cell entry.

**Fig 3:**
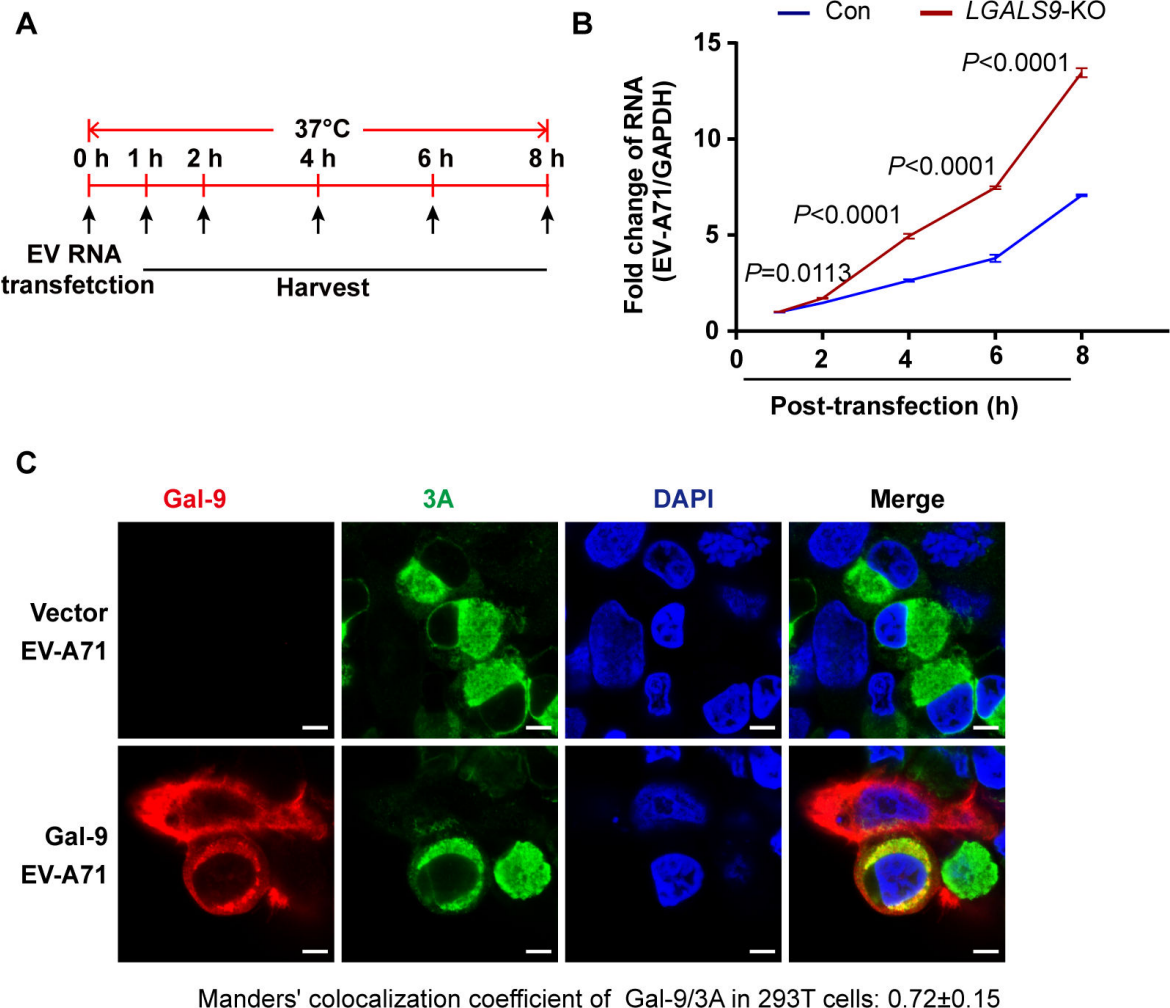
Gal-9 restricted EV-A71 replication. (**A**) Schematic of EV-A71 RNA transinfection in *LGALS9*-KO and control A549 cells. The full-genome RNA of EV-A71 was transcribed *in vitro* and purified viral RNA was transfected into *LGALS9*-KO and control A549 cells. After transfection, the cells were incubated at 37°C and collected at the indicated times. (**B**) Viral RNAs were detected by using qRT-PCR. The experiment was done at least twice, and one representative is shown. Error bars indicate SD of technical triplicates. *P*-values of the two-tailed Student’s *t*-test are indicated. (**C**) 293T cells were transfected with a vector or isoform Δ5 of Gal-9-Flag expression plasmid. At 24 h post-transfection, the cells were infected with 1 MOI EV-A71 for 12 h and then immunostained with the indicated antibodies. Scale bar, 5 μm. Colocalization between Gal-9 and 3A of the Manders’ colocalization coefficient above auto-threshold was indicated (at least 10 cells from two independent experiments).

To determine whether Gal-9 affects RNA replication directly, we bypassed the entry step by transfecting purified full-length EV-A71 RNA into wild-type and *LGALS9*-KO cells (Fig. 3A). qRT-PCR analysis revealed a significant increase in viral RNA in *LGALS9*-KO cells just 2 h post-transfection (Fig. 3B), suggesting that Gal-9 restricts EV-A71 RNA replication independently of entry. To determine if Gal-9 localizes to sites of viral RNA synthesis, we performed immunofluorescence in EV-A71-infected 293T cells expressing Gal-9-Flag. Immunofluorescence showed co-localization of Gal-9 with the viral 3A protein, which is known to localize to replication sites (38) (Fig. 3C). This finding supports that Gal-9 exerts its antiviral effect at viral replication sites.

### Gal-9 targets viral VP2 protein

**Fig 4:**
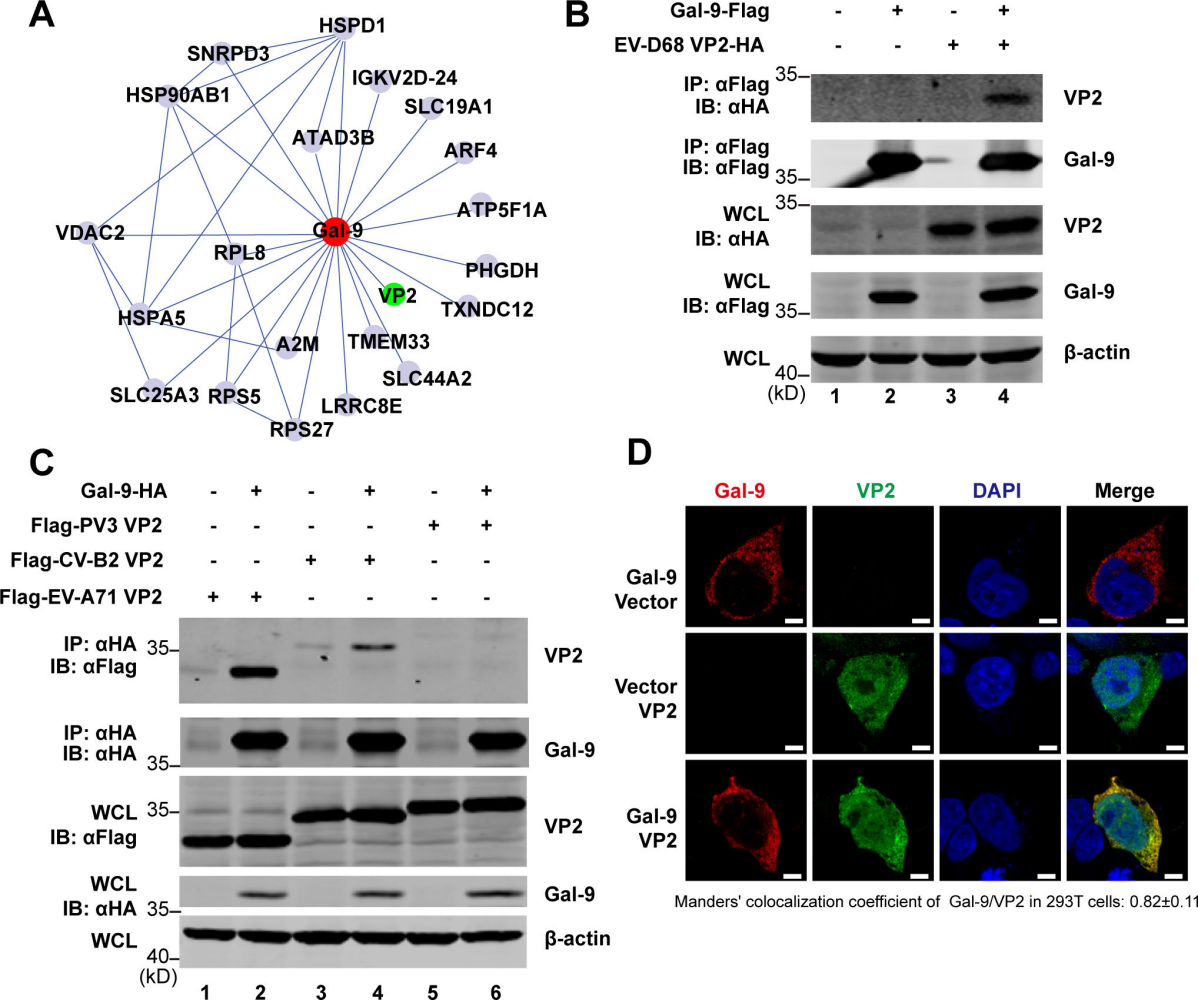
Gal-9 interacted with VP2 of EV-D68, EV-A71, but not PV3. (**A**) Protein–protein interactions of Gal-9 with host factors (gray) or viral protein (green) after EV-D68 infection are shown. (**B**) 293T cells were transfected with a vector, isoform Δ5 of Gal-9-Flag and EV-D68 VP2-HA. Cell lysates were IP with anti-Flag antibody. Immunoprecipitates and aliquots of cell lysates were subjected to WB with antibodies against Flag, HA, and β-actin. (**C**) 293T cells were transfected with a vector, isoform Δ5 of Gal-9-HA and Flag-EV-A71 VP2, Flag-CV-B2 VP2, or Flag-PV3 VP2. Cell lysates were immunoprecipitated (IP) with anti-HA antibody. Immunoprecipitates and aliquots of cell lysates were subjected to WB with antibodies against Flag, HA, and β-actin. (**D**) 293T cells were transfected with isoform Δ5 of Gal-9-HA and/or Flag-EV-A71 VP2 expression plasmid. At 24 h post-transfection, the cells were immunostained with the indicated antibodies. Scale bar, 5 μm. Colocalization between Gal-9 and VP2 using the Manders’ colocalization coefficient above auto-threshold was indicated (at least 10 cells from two independent experiments).

To gain further mechanistic insights into Gal-9’s antiviral function, we investigated whether Gal-9 interacts with viral proteins. Immunoprecipitation-mass spectrometry (IP-MS) analysis was performed in EV-D68-infected cells to identify Gal-9-associated proteins. Among the viral proteins, only VP2 was found to be associated with Gal-9 (Fig. 4A). Co-immunoprecipitation (co-IP) assays confirmed this interaction (Fig. 4B). We further examined whether Gal-9 interacted with VP2 from several other EVs, including EV-A71, CV-B2, and PV3. Co-IP experiments suggested that Gal-9 interacted with VP2 of EV-A71 and CV-B2, but not with VP2 of PV3 (Fig. 4C). Notably, PV3 was the only tested virus whose replication was unaffected by Gal-9 depletion (Fig. 1C and D), suggesting that Gal-9 targets VP2 to exert its antiviral effects. To confirm the interaction between EV-A71 VP2 and Gal-9, we carried out immunofluorescence analysis. As shown in Fig. 4D, exogenously overexpressed VP2 and Gal-9 co-localized in the cytoplasm.

**Fig 5:**
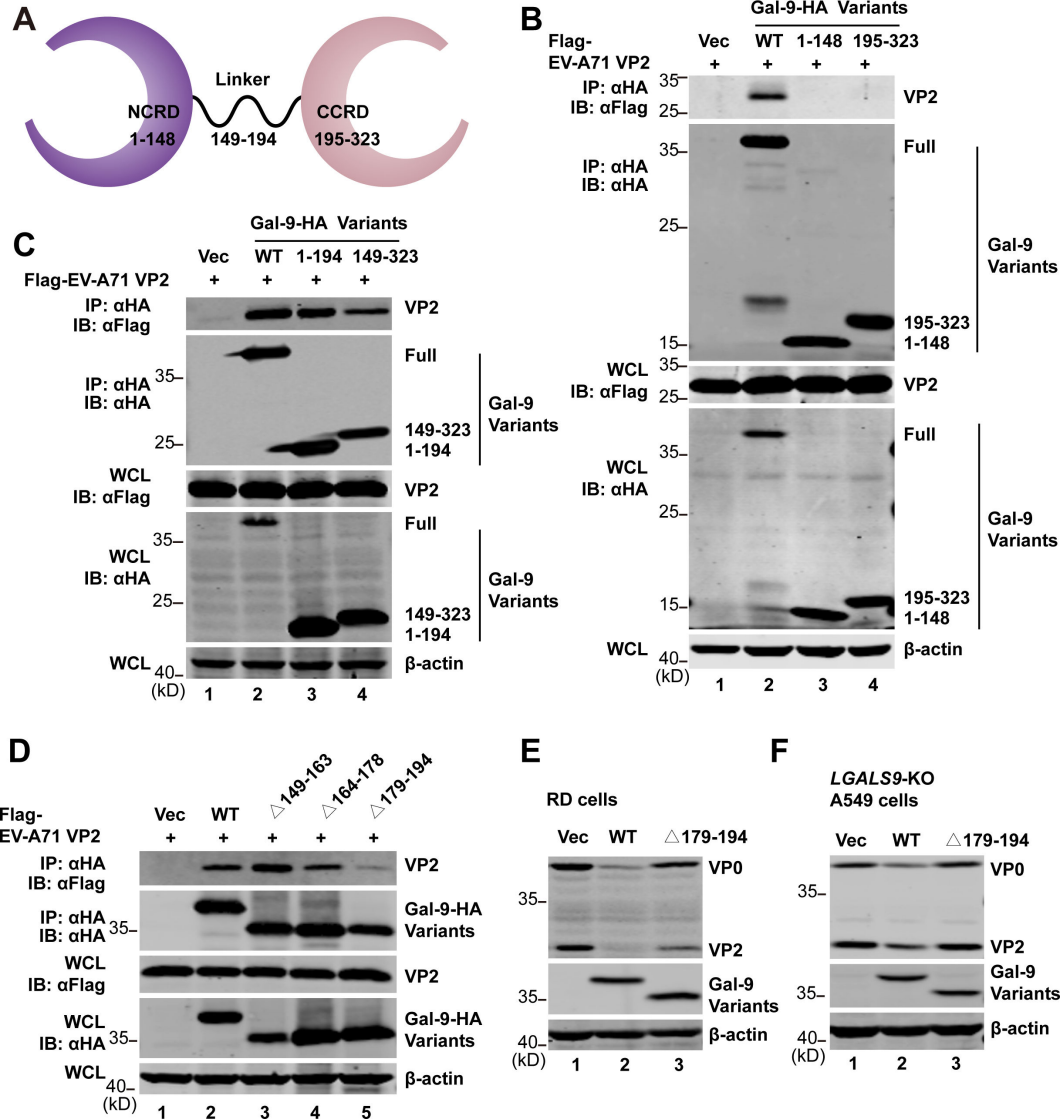
Gal-9 interacted with VP2 to restrict EVs replication. (**A**) Schematic of the isoform Δ5 of Gal-9, which contains an NCRD domain, a CCRD domain, and a linker region. (**B**) 293T cells were transfected with a vector, Flag-EV-A71 VP2, and Gal-9-HA, NCRD (1–148), or CCRD (195–323) without the linker region. Cell lysates were IP with anti-HA antibody. Immunoprecipitates and aliquots of cell lysates were subjected to WB with antibodies against Flag, HA, and β-actin. (**C**) 293T cells were transfected with a vector, Flag-EV-A71 VP2, and Gal-9-HA, NCRD, or CCRD with the linker region. Cell lysates were IP with anti-HA antibody. Immunoprecipitates and aliquots of cell lysates were subjected to WB with antibodies against Flag, HA, and β-actin. (**D**) 293T cells were transfected with a vector, Flag-EV-A71 VP2, and Gal-9-HA or truncation mutants. Cell lysates were IP with anti-HA antibody. Immunoprecipitates and aliquots of cell lysates were subjected to WB with antibodies against Flag, HA, and β-actin. (**E**) RD cells were transfected with a vector, Gal-9-HA, or Δ179–194 truncation mutant for 24 h. Cells were then infected with 0.5 MOI of EV-A71. At 24 h after infection, cell lysates were analyzed by WB using antibodies against HA, VP0/VP2, and β-actin. (**F**) *LGALS9*-KO A549 cells were transfected with a vector, Gal-9-HA, or Δ179–194 truncation mutant for 24 h. Cells were then infected with 3 MOI of EV-A71. At 24 h after infection, cell lysates were analyzed by WB using antibodies against HA, VP0/VP2, and β-actin.

According to the X-ray structure, the Δ5 isoform of Gal-9 (GenBank accession no. NM_002308) consists of an N-terminal carbohydrate recognition domain (NCRD, residues 1–148) and a C-terminal CRD (CCRD, residues 195–323). These two domains are connected by a flexible peptide linker (residues 149–194) (39) (Fig. 5A). To map the Gal-9 region responsible for VP2 binding, we constructed different truncation variants of Gal-9 based on this information and detected their interactions with EV-A71 VP2 by co-IP assays. The results showed that neither the NCRD nor the CCRD domain interacted with viral VP2 (Fig. 5B). However, adding the linker region to the C-terminus of the NCRD domain or the N-terminus of the CCRD domain enabled VP2 binding (Fig. 5C), indicating that the linker region of Gal-9 is essential for its interaction with VP2. Furthermore, functional sites in the linker region were fine-mapped. As shown in Fig. 5D, the deletion of amino acids from positions 179 to 194 (Δ179–194) of the linker region significantly disrupted the interaction between Gal-9 and VP2, whereas deletions at 149–163 (Δ149–163) or 164–178 (Δ164–178) had no effect. Consistently, the Δ179–194 mutant of Gal-9 almost completely lost its antiviral activity against EV-A71 in both RD (Fig. 5E) and *LGALS9*-KO A549 cells (Fig. 5F). Collectively, these results suggest that the interaction between Gal-9 and VP2, mediated by the linker region of Gal-9, is critical for its antiviral activity against enteroviruses.

### *LGALS9* deficiency increases viral pathogenicity in mice

**Fig 6:**
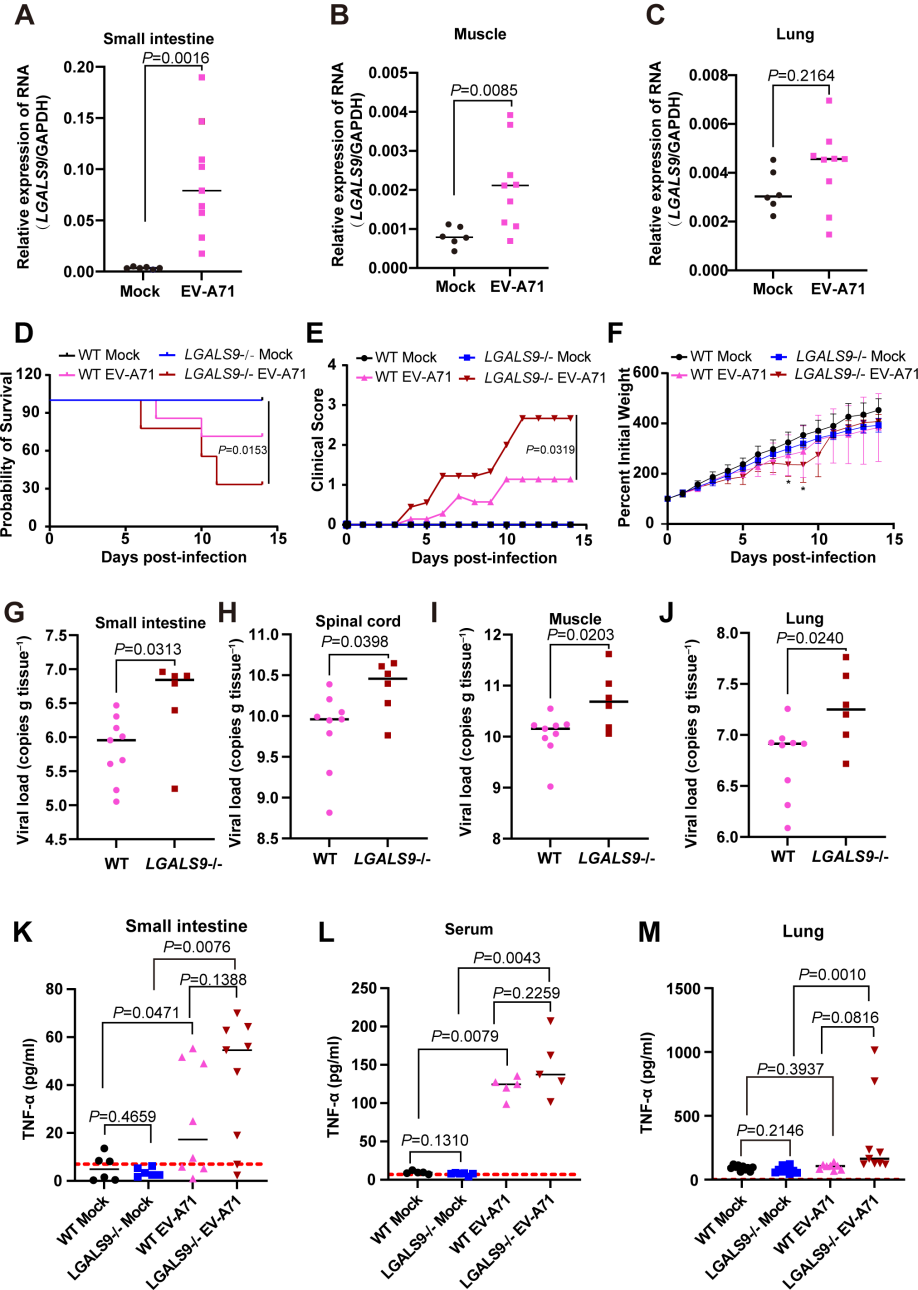
Gal-9 is important for EV-A71 infection in mice. Three-day-old suckling WT and *LGALS9*^−/−^ mice were challenged with 3.5 × 10^6^ pfu EV-A71 (strain SHAPHC 695F) or Dulbecco’s modified Eagle’s medium by intraperitoneal route. The mRNA levels of *LGALS9* in mock or EV-A71-infected mice were detected using qRT-PCR in the small intestine (**A**), hind-limb skeletal muscle (**B**), and lung (**C**) at 5 days post-infection. The survival rates (**D**), clinical scores (**E**), and body weights (**F**) of the infected mice were monitored. * Compared to the mock-infected group, *LGALS9*^−/−^ mice with EV-A71 infection exhibited delayed weight gain at 8 and 9 days post-infection. *P* = 0.0126 and *P* = 0.0157. The viral load of EV-A71 present in the small intestine (**G**), spinal cord (**H**), hind-limb skeletal muscle (**I**), and lung (**J**) at 5 days post-infection of the indicated mice was detected using qRT-PCR. Concentrations of TNF-α in the small intestine (**K**), serum (**L**), and lung (**M**) of EV-A71- or mock-infected 3-day-old suckling WT and *LGALS9*^−/−^ mice were determined by ELISA. The samples were collected at 5 days post-infection. The red horizontal dotted line indicates the limit of detection for this assay (7 pg/mL).

To further investigate the role of Gal-9 in EV pathogenesis, we used a neonatal mouse model of EV-A71 infection. Intraperitoneal injection of EV-A71 into neonatal mice established a model that mimics EV pathogenesis, leading to systemic viral disease characterized by high viral loads in the muscle and intestine, limb paralysis, and mortality (40, 41). We detected *LGALS9* expression in the tissues of EV-A71-infected mice by qRT-PCR analysis. Compared to mock-infected mice, *LGALS9* expression was significantly elevated in the small intestine and skeletal muscle of EV-A71-infected mice (Fig. 6A and B). Although *LGALS9* expression also increased in the lungs following EV-A71 infection, the difference was not statistically significant (Fig. 6C).

To investigate the role of Gal-9 in EV infection *in vivo*, we used *LGALS9* knockout mice. Homozygous *LGALS9* null (*LGALS9*^−/−^) mice were viable and exhibited no apparent developmental defects. When 3-day-old suckling mice were injected with the same dose of EV-A71, *LGALS9*^−/−^ mice showed lower survival rates (Fig. 6D) and significantly higher clinical scores (Fig. 6E) than WT mice. Additionally, *LGALS9*^−/−^ mice exhibited impaired weight gain at 8 and 9 days post-infection (Fig. 6F). These results confirm that *LGALS9* deficiency exacerbates EV-A71 pathogenicity. To assess viral replication, we measured EV-A71 viral load in various tissues on day 5 post-infection. Compared with WT mice, *LGALS9*^−/−^ mice exhibited significantly higher viral load in the small intestines, spinal cord, skeletal muscle, and lungs (Fig. 6G–J). These results demonstrated that *LGALS9* deficiency enhances EV-A71 replication and pathogenesis in mice.

Considering the pro-inflammatory characteristics of EV-A71, we assessed TNF-α levels in EV-A71-infected mice by measuring their concentrations. In uninfected mice, *LGALS9* knockout did not affect the concentrations of TNF-α in the small intestine, serum, and lungs (Fig. 6K through M, mock). EV-A71 infection led to a significant increase in TNF-α concentration, especially in *LGALS9*-deficient mice (Fig. 6K through M). These results suggest that the higher pathogenicity in *LGALS9*-deficient mice results from enhanced EV-A71 replication, not a reduced immune response.

## DISCUSSION

The role of Gal-9 in EV infection has not been previously reported. Compared to mock-infected mice, *LGALS9* expression was also significantly higher in the small intestine and skeletal muscle of EV-A71-infected mice. EV-A71 was more effective in *LGALS9*^−/−^ mice than in WT mice. EV-A71 resulted in more limb paralysis and death in *LGALS9*^−/−^ mice than in WT mice. These results suggest that Gal-9 plays an important role in the host response to EV infection.

Gal-9 regulates immunity and inflammation by regulating the function of NK cells, monocytes, and T cells. The absence of Gal-9 influences the immune response of these cells. Compared to control mice, NK cell levels were similar but demonstrated greater activity in the spleens and livers of Gal-9 KO mice (42). In a zymosan-induced peritonitis model, neutrophil recruitment was significantly reduced in Gal-9 knockout mice (43). Although Gal-9 deficiency resulted in altered function of these cells, LPS administration did not exacerbate the significant differences in TNF-α and IL-6 levels between WT and Gal-9 KO mice (44). Similarly, EV-A71 infection induced a significant increase in TNF-α levels in mice, which is consistent with higher levels of inflammatory mediators in severe HFMD cases (45), but no statistically significant difference was observed between WT and Gal-9 KO mice (Fig. 6). Therefore, we infer that the higher pathogenicity in *LGALS9*-deficient mice results from enhanced EV-A71 replication and not a reduced immune response.

Gal-9 is a tandem repeat-type galectin with two distinct CRDs connected by a flexible peptide linker (46, 47). The linker region not only influences the rotational freedom of the CRDs but also plays an important role in protein–protein interactions (48). In this study, we found that Gal-9 interacts with VP2, and this interaction is dependent on the linker region (amino acid sequence: PAIPPMMYPHPAYPMP), not the CRDs. This sequence is present in all six splice variants of Gal-9, which explains why all isoforms exhibit anti-EV-A71 activity (Fig. 1F). When the interaction region was deleted, the antiviral effect of Gal-9 diminished. Gal-9 has been reported to degrade HBV core protein and NLRP3 by autophagy to limit viral replication and inflammation (29, 34). We did not observe VP2 degradation when co-transfected with Gal-9. These results suggest that the antiviral mechanism exerted by Gal-9 in EVs depends on its interaction with VP2.

VP2 plays an important role in EVs pathogenesis and proliferation. VP2 was shown to play a role during the entry stage by interacting with the SCARB2 receptor, likely playing a role during the uncoating step of the entry stage (49). A point mutation in VP2 can markedly alter the ability of CV-B3 to induce myocarditis and EV-A71 neurotropism in mice (50, 51). VP2 degradation through neddylation suppresses EV-A71 multiplication (52). Small interfering RNAs targeting VP2 in EV-A71 and RV-16 can effectively block viral replication (53, 54). VP2 protein of Senecavirus A, foot-and-mouth disease virus, and EV-A71 regulates IKBKE and inhibits the type I IFN signaling pathway to promote viral replication (55). The mechanism by which VP2 is involved in the RNA replication of EVs is unclear. The capsid protein of the flavivirus dengue virus accumulates on the surface of lipid droplets in infected cells and is dispensable for RNA synthesis (56). In EV-infected cells, lipid droplets provide fatty acids for replication compartment biogenesis (57–59). Except for 2BC, other PV proteins, including the intermediate proteins 3CD, 3C, 3D, and capsid proteins, were not localized to lipid droplets (58). Whether VP2 of EV-A71, EV-D68, and CV-B2 is located in lipid droplets requires further investigation. Further investigations are required to determine whether VP2 interacts with other viral or host proteins that may be involved in viral replication.

In conclusion, this study revealed that Gal-9 plays an important role in host antiviral response to EV infection. Gal-9 inhibited multiple EVs replication through interaction with VP2, which was demonstrated by experiments showing that Gal-9 truncations lacking VP2-binding capacity failed to suppress enterovirus replication in *LGALS9*-KO A549 cells. Further understanding of the role and mechanism of Gal-9 in viral infection may lead to the identification of novel therapeutic targets.

## MATERIALS AND METHODS

### Cell lines and viruses

Human 293T (ATCC, #CCL-11268) and RD (ATCC, #CCL-136) cells were purchased from the ATCC. *LGALS9* knockout and control A549 cells were purchased from Edigene, Inc. These cells were cultured in Dulbecco’s modified Eagle’s medium (Invitrogen, Carlsbad, CA) supplemented with 10% heat-inactivated fetal bovine serum (FBS) (HyClone, Logan, UT), 100 U/mL penicillin, and 100 U/mL streptomycin at 37°C in a 5% CO_2_ humidified atmosphere. Human monocytic THP-1 (TIB-202; ATCC) cells were cultured in RPMI 1640 medium supplemented with 10% FBS. *LGALS9*-knockout THP-1 cells were generated using CRISPR-Cas9 technology (60) with a guide RNA targeting the sequence CACCGAGAGCAGTTACGGGAAATGG. Low-passage cells after direct purchase from ATCC were used, and all cells were tested for mycoplasma-free status. EV-A71 (strain Fuyang-0805, isolated in 2008, GenBank accession no. FJ439769), CV-B2 (GenBank accession No. KM386639), and EV-D68 (GenBank accession No. KT285485) were used as previously described (7, 38, 61). Human poliovirus 3 strain Sabin 3 (GenBank accession no. AY184221) is a vaccine strain provided by Professor Fang Huang (Beijing Center for Disease Prevention and Control). The EV-A71 strain SHAPHC695F (GenBank accession no. JQ736684) was isolated in 2010 and could induce paralytic symptoms in suckling mice (40, 41). SHAPHC695F and the Fuyang-0805 strain both belong to the C4a subtype, with 99.5% amino acid homology between them.

### Plasmids and antibodies

The Gal-9 plasmid was purchased from OriGene. A Flag-tag or HA-tag was constructed at the C-terminus. The mutated variants of Gal-9-tagged HA were constructed using a Site-Directed Mutagenesis Kit (Stratagene, La Jolla, CA). All variants were confirmed by sequencing. Flag-EV-A71 VP2, Flag-CV-B2 VP2, and Flag-PV3 VP2 were amplified from RNA of the viruses and cloned into the pEGFP-C1 expression vector with the Flag-tag replacing GFP at the N-terminus.

The antibodies used in this research were as follows: Flag antibody from Sigma-Aldrich (1:4,000, Cat# F3165), β-actin antibody from Sigma-Aldrich (1:4,000, Cat# A5441), HA antibody from Sigma-Aldrich (1:10,000, Cat# H9658), anti-galectin-9 from R&D Systems (1:250, Cat# MAB20453), EV-D68 VP1 antibody from GeneTex (1:1,000, Cat# GTX132313), anti-EV-A71 (1:4,000, Cat# MAB979), anti-CV-B2 (1:1,000, Cat# MAB946) from Sigma-Aldrich, anti-Poliovirus (type 1–3) antibody (1:2,000, Cat# ab22450) from Abcam, anti-dsRNA J2 (1:1,000, Cat# 10,020,100) from Scicons, Enterovirus 71 3D antibody (1:1,000, Cat# GTX630193) from GeneTex. IRDye 800-labeled IgG and IRDye 680-labeled IgG secondary antibodies were purchased from Li-Cor Biosciences (Lincoln, NE, USA).

### *In vitro* transcription and purified viral RNA transfection

EV-A71 cDNA constructs were digested with MluI to produce a linear DNA template, purified by phenol/chloroform extraction and ethanol precipitation, and then dissolved in RNase-free water (62). For the *in vitro* RNA transcription reaction, 1 μg of the linear DNA template was transcribed using the MEGAscript T7 Transcription Kit in a total volume of 20 μL. The *in vitro* transcripts were purified using phenol/chloroform and dissolved in RNase-free water. Two micrograms of transcribed RNA was transfected into 2 × 10^5^ A549 cells using DMRIE-C.

### Real-time PCR

Total RNA was extracted using the TRIzol reagent (Invitrogen) and reverse-transcribed using the SuperScript cDNA synthesis kit (Invitrogen). Real-time quantitative PCR was performed using a SYBR Green Kit (Takara Bio) with a 20 μL mixture containing 1 μL cDNA, 10 μL 2 × TB Green Premix Ex Taq (Takara), and 500 nM of each primer (38, 63) on a CFX Optics Module System (Bio-Rad). The optimized thermal cycling parameters were 95°C for 3 min, 45 cycles of 95°C for 5 s and 60°C for 30 s. Melting curves were generated by increasing the temperature from 65°C to 95°C at 0.5°C/s. The relative gene expression was normalized to that of GAPDH in the same sample using the 2^−ΔCT^ or 2^−ΔΔCT^ method.

### Immunofluorescence

Cells were washed with PBS and fixed with 4% paraformaldehyde. Then, the cells were permeabilized with 0.5% Triton X-100. After the cells were washed with PBS, they were blocked and stained with primary antibodies, then stained with Alexa Fluor 488- and 594-conjugated secondary antibodies. Nuclei were stained with DAPI (Sigma). Fluorescence images were obtained and analyzed using a laser scanning confocal microscope (Leica TCS SP8 and Zeiss LSM 800).

### Immunoprecipitation

Transfected cells were lysed with RIPA, containing protease inhibitor cocktail (Roche, Indianapolis, IN). Lysates of cells were incubated with primary antibodies in 500 μL RIPA buffer at 4°C overnight on a rotator in the presence of protein A/G agarose beads (Santa Cruz Biotechnology, Santa Cruz, CA). Immunocomplexes captured on the protein A/G agarose were fractionated by 12% SDS-PAGE and transferred to nitrocellulose membranes for analysis.

### Viral infection and immunoprecipitations for MS analysis

293T cells were transfected with vector or Gal-9-Flag plasmid. After 24 h post-transfection, cells were mock-infected or infected with EV-D68 virus at 0.5 MOI. After infection for 8 h, the above cells were lysed for immunoprecipitation using ANTI-FLAG M2 Affinity Gel (Millipore, Cat# A2220). The immunoprecipitated proteins were confirmed using western blot, and the remaining material was immediately used for mass spectrometry analysis.

The identified proteins were manually examined and filtered, and those from the vector and mock-infected groups were used as controls to remove redundant proteins and identify specific interacting proteins. A protein network was generated using the STRING tool in Cytoscape (v.3.9.1), after entering the candidates.

### EV-A71 infection in mice

Heterozygous C57BL/6N mice carrying the *LGALS9* null allele were purchased from Cyagen Biosciences. The mice were then bred and housed at a specific-pathogen-free laboratory animal facility. Homozygous (*LGALS9*^−/−^) and wild-type mice were identified according to the protocol provided by Cyagen Biosciences.

Three-day-old suckling mice (2 ± 0.2 g) were challenged with 3.5 × 10^6^ pfu EV-A71 (strain SHAPHC695F) or Dulbecco’s modified Eagle’s medium by intraperitoneal route (41). The weights, symptoms, and survival rates of the infected mice were monitored daily for 15 days.

The sickness of the mice was evaluated using a graded score (0, healthy; 1, paralysis in a single limb; 2, paralysis in two limbs; 3, paralysis in four limbs; and 4, death). The scores of the individual mice were weighted and summed to obtain a combined disease severity score for each group.

TNF-α levels in the blood, lung, and small intestine of the mice were quantified using ELISA (ExCell Bio; Cat# EM 008) according to the manufacturer’s instructions. The concentrations of TNF-α in the samples were calculated using a standard curve.

### Statistics

Two-group comparisons were performed using a two-tailed Student’s *t*-test. The values *P* < 0.05 (*), *P* < 0.01 (**), and *P* < 0.001 (***) were considered significant.

## Data Availability

All data from this study are included in the paper and are available from the corresponding author upon reasonable request.

## References

[1] Fischer TK, Simmonds P, Harvala H. 2022. The importance of enterovirus surveillance in a post-polio world. Lancet Infect Dis 22:e35–e40. doi:10.1016/S1473-3099(20)30852-5 34265258

[2] Midgley CM, Watson JT, Nix WA, Curns AT, Rogers SL, Brown BA, Conover C, Dominguez SR, Feikin DR, Gray S, et al.. 2015. Severe respiratory illness associated with a nationwide outbreak of enterovirus D68 in the USA (2014): a descriptive epidemiological investigation. Lancet Respir Med 3:879–887. doi:10.1016/S2213-2600(15)00335-5 26482320 PMC5693332

[3] Gilrane VL, Zhuge J, Huang W, Nolan SM, Dhand A, Yin C, Salib C, Shakil F, Engel H, Fallon JT, Wang G. 2020. Biennial upsurge and molecular epidemiology of enterovirus D68 infection in New York, USA, 2014 to 2018. J Clin Microbiol 58:e00284-20. doi:10.1128/JCM.00284-20 32493783 PMC7448634

[4] Kramer R, Sabatier M, Wirth T, Pichon M, Lina B, Schuffenecker I, Josset L. 2018. Molecular diversity and biennial circulation of enterovirus D68: a systematic screening study in Lyon, France, 2010 to 2016. Euro Surveill 23:1700711. doi:10.2807/1560-7917.ES.2018.23.37.1700711 30229724 PMC6144471

[5] Andres C, Vila J, Creus-Costa A, Pinana M, Gonzalez-Sanchez A, Esperalba J, Codina MG, Castillo C, Martin MC, Fuentes F, Rubio S, Garcia-Comunas K, Vasquez-Mercado R, Saubi N, Rodrigo C, Pumarola T, Anton A. 2022. Enterovirus D68 in hospitalized children, Barcelona, Spain, 2014-2021. Emerg Infect Dis 28:1327–1331. doi:10.3201/eid2807.220264 35731133 PMC9239859

[6] Messacar K, Pretty K, Reno S, Dominguez SR. 2019. Continued biennial circulation of enterovirus D68 in Colorado. J Clin Virol 113:24–26. doi:10.1016/j.jcv.2019.01.008 30825833

[7] Xiang Z, Xie Z, Liu L, Ren L, Xiao Y, Paranhos-Baccalà G, Wang J. 2016. Genetic divergence of enterovirus D68 in China and the United States. Sci Rep 6:27800. doi:10.1038/srep27800 27278628 PMC4899779

[8] Nayak G, Bhuyan SK, Bhuyan R, Sahu A, Kar D, Kuanar A. 2022. Global emergence of enterovirus 71: a systematic review. Beni Suef Univ J Basic Appl Sci 11:78. doi:10.1186/s43088-022-00258-4 35730010 PMC9188855

[9] Zhou T, Hu H, Gao J, Yu H, Jit M, Wang P. 2024. Health-related quality of life and economic burden among hospitalized children with hand, foot, and mouth disease: a multiregional study in China. Pharmacoecon Open 8:459–469. doi:10.1007/s41669-023-00468-1 38195850 PMC11058149

[10] Baggen J, Thibaut HJ, Strating JRPM, van Kuppeveld FJM. 2018. The life cycle of non-polio enteroviruses and how to target it. Nat Rev Microbiol 16:368–381. doi:10.1038/s41579-018-0005-4 29626210

[11] Huang SW, Wang YF, Yu CK, Su IJ, Wang JR. 2012. Mutations in VP2 and VP1 capsid proteins increase infectivity and mouse lethality of enterovirus 71 by virus binding and RNA accumulation enhancement. Virology (Auckl) 422:132–143. doi:10.1016/j.virol.2011.10.015 22078110

[12] Liu FT, Stowell SR. 2023. The role of galectins in immunity and infection. Nat Rev Immunol 23:479–494. doi:10.1038/s41577-022-00829-7 36646848 PMC9842223

[13] Yang RY, Rabinovich GA, Liu FT. 2008. Galectins: structure, function and therapeutic potential. Expert Rev Mol Med 10:e17. doi:10.1017/S1462399408000719 18549522

[14] Lee PH, Liu CM, Ho TS, Tsai YC, Lin CC, Wang YF, Chen YL, Yu CK, Wang SM, Liu CC, Shiau AL, Lei HY, Chang CP. 2015. Enterovirus 71 virion-associated galectin-1 facilitates viral replication and stability. PLoS One 10:e0116278. doi:10.1371/journal.pone.0116278 25706563 PMC4338065

[15] Huang WC, Chen HL, Chen HY, Peng KP, Lee Y, Huang LM, Chang LY, Liu FT. 2016. Galectin-3 and its genetic variation rs4644 modulate enterovirus 71 infection. PLoS One 11:e0168627. doi:10.1371/journal.pone.0168627 28002441 PMC5176291

[16] Heusschen R, Schulkens IA, van Beijnum J, Griffioen AW, Thijssen VL. 2014. Endothelial LGALS9 splice variant expression in endothelial cell biology and angiogenesis. Biochimica et Biophysica Acta (BBA) - Molecular Basis of Disease 1842:284–292. doi:10.1016/j.bbadis.2013.12.003 24333696

[17] Moar P, Tandon R. 2021. Galectin-9 as a biomarker of disease severity. Cell Immunol 361:104287. doi:10.1016/j.cellimm.2021.104287 33494007

[18] Wada J, Kanwar YS. 1997. Identification and characterization of galectin-9, a novel beta-galactoside-binding mammalian lectin. J Biol Chem 272:6078–6086. doi:10.1074/jbc.272.9.6078 9038233

[19] Wada J, Ota K, Kumar A, Wallner EI, Kanwar YS. 1997. Developmental regulation, expression, and apoptotic potential of galectin-9, a beta-galactoside binding lectin. J Clin Invest 99:2452–2461. doi:10.1172/JCI119429 9153289 PMC508086

[20] Nagae M, Nishi N, Murata T, Usui T, Nakamura T, Wakatsuki S, Kato R. 2006. Crystal structure of the galectin-9 N-terminal carbohydrate recognition domain from Mus musculus reveals the basic mechanism of carbohydrate recognition. J Biol Chem 281:35884–35893. doi:10.1074/jbc.M606648200 16990264

[21] Türeci O, Schmitt H, Fadle N, Pfreundschuh M, Sahin U. 1997. Molecular definition of a novel human galectin which is immunogenic in patients with Hodgkin’s disease. J Biol Chem 272:6416–6422. doi:10.1074/jbc.272.10.6416 9045665

[22] Li Y, Feng J, Geng S, Geng S, Wei H, Chen G, Li X, Wang L, Wang R, Peng H, Han G, Shen B, Li Y. 2011. The N- and C-terminal carbohydrate recognition domains of galectin-9 contribute differently to its multiple functions in innate immunity and adaptive immunity. Mol Immunol 48:670–677. doi:10.1016/j.molimm.2010.11.011 21146220

[23] Hirashima M, Kashio Y, Nishi N, Yamauchi A, Imaizumi T, Kageshita T, Saita N, Nakamura T. 2002. Galectin-9 in physiological and pathological conditions. Glycoconj J 19:593–600. doi:10.1023/B:GLYC.0000014090.63206.2f 14758084

[24] Matsuura A, Tsukada J, Mizobe T, Higashi T, Mouri F, Tanikawa R, Yamauchi A, Hirashima M, Tanaka Y. 2009. Intracellular galectin-9 activates inflammatory cytokines in monocytes. Genes Cells 14:511–521. doi:10.1111/j.1365-2443.2009.01287.x 19335620

[25] Dai SY, Nakagawa R, Itoh A, Murakami H, Kashio Y, Abe H, Katoh S, Kontani K, Kihara M, Zhang SL, Hata T, Nakamura T, Yamauchi A, Hirashima M. 2005. Galectin-9 induces maturation of human monocyte-derived dendritic cells. J Immunol 175:2974–2981. doi:10.4049/jimmunol.175.5.2974 16116184

[26] Kadowaki T, Arikawa T, Shinonaga R, Oomizu S, Inagawa H, Soma G, Niki T, Hirashima M. 2012. Galectin-9 signaling prolongs survival in murine lung-cancer by inducing macrophages to differentiate into plasmacytoid dendritic cell-like macrophages. Clin Immunol 142:296–307. doi:10.1016/j.clim.2011.11.006 22177847

[27] Kashio Y, Nakamura K, Abedin MJ, Seki M, Nishi N, Yoshida N, Nakamura T, Hirashima M. 2003. Galectin-9 induces apoptosis through the calcium-calpain-caspase-1 pathway. J Immunol 170:3631–3636. doi:10.4049/jimmunol.170.7.3631 12646627

[28] Seki M, Oomizu S, Sakata KM, Sakata A, Arikawa T, Watanabe K, Ito K, Takeshita K, Niki T, Saita N, Nishi N, Yamauchi A, Katoh S, Matsukawa A, Kuchroo V, Hirashima M. 2008. Galectin-9 suppresses the generation of Th17, promotes the induction of regulatory T cells, and regulates experimental autoimmune arthritis. Clin Immunol 127:78–88. doi:10.1016/j.clim.2008.01.006 18282810

[29] Wang W, Qin Y, Song H, Wang L, Jia M, Zhao C, Gong M, Zhao W. 2021. Galectin-9 targets NLRP3 for autophagic degradation to limit inflammation. J Immunol 206:2692–2699. doi:10.4049/jimmunol.2001404 33963043

[30] Reddy PBJ, Sehrawat S, Suryawanshi A, Rajasagi NK, Mulik S, Hirashima M, Rouse BT. 2011. Influence of galectin-9/Tim-3 interaction on herpes simplex virus-1 latency. J Immunol 187:5745–5755. doi:10.4049/jimmunol.1102105 22021615 PMC3221893

[31] Machala EA, Avdic S, Stern L, Zajonc DM, Benedict CA, Blyth E, Gottlieb DJ, Abendroth A, McSharry BP, Slobedman B. 2019. Restriction of human cytomegalovirus infection by galectin-9. J Virol 93:e01746-18. doi:10.1128/JVI.01746-18 30487283 PMC6340044

[32] Nebbia G, Peppa D, Schurich A, Khanna P, Singh HD, Cheng Y, Rosenberg W, Dusheiko G, Gilson R, ChinAleong J, Kennedy P, Maini MK. 2012. Upregulation of the Tim-3/galectin-9 pathway of T cell exhaustion in chronic hepatitis B virus infection. PLoS One 7:e47648. doi:10.1371/journal.pone.0047648 23112829 PMC3480425

[33] Mostafavi S, Yoshida H, Moodley D, LeBoité H, Rothamel K, Raj T, Ye CJ, Chevrier N, Zhang S-Y, Feng T, Lee M, Casanova J-L, Clark JD, Hegen M, Telliez J-B, Hacohen N, De Jager PL, Regev A, Mathis D, Benoist C. 2016. Parsing the interferon transcriptional network and its disease associations. Cell 164:564–578. doi:10.1016/j.cell.2015.12.032 26824662 PMC4743492

[34] Miyakawa K, Nishi M, Ogawa M, Matsunaga S, Sugiyama M, Nishitsuji H, Kimura H, Ohnishi M, Watashi K, Shimotohno K, Wakita T, Ryo A. 2022. Galectin-9 restricts hepatitis B virus replication via p62/SQSTM1-mediated selective autophagy of viral core proteins. Nat Commun 13:531. doi:10.1038/s41467-022-28171-5 35087074 PMC8795376

[35] Bozorgmehr N, Mashhouri S, Perez Rosero E, Xu L, Shahbaz S, Sligl W, Osman M, Kutsogiannis DJ, MacIntyre E, O’Neil CR, Elahi S. 2021. Galectin-9, a player in cytokine release syndrome and a surrogate diagnostic biomarker in SARS-CoV-2 infection. mBio 12:e00384-21. doi:10.1128/mBio.00384-21 33947753 PMC8262904

[36] Du L, Bouzidi MS, Gala A, Deiter F, Billaud J-N, Yeung ST, Dabral P, Jin J, Simmons G, Dossani ZY, Niki T, Ndhlovu LC, Greenland JR, Pillai SK. 2023. Human galectin-9 potently enhances SARS-CoV-2 replication and inflammation in airway epithelial cells. J Mol Cell Biol 15. doi:10.1093/jmcb/mjad030 PMC10668544 37127426

[37] Smith CD, Craft DW, Shiromoto RS, Yan PO. 1986. Alternative cell line for virus isolation. J Clin Microbiol 24:265–268. doi:10.1128/jcm.24.2.265-268.1986 3018038 PMC268886

[38] Xiao X, Lei X, Zhang Z, Ma Y, Qi J, Wu C, Xiao Y, Li L, He B, Wang J. 2017. Enterovirus 3A facilitates viral replication by promoting phosphatidylinositol 4-kinase IIIβ-ACBD3 interaction. J Virol 91:e00791-17. doi:10.1128/JVI.00791-17 28701404 PMC5599760

[39] Yoshida H, Nishi N, Wada K, Nakamura T, Hirashima M, Kuwabara N, Kato R, Kamitori S. 2017. X-ray structure of a protease-resistant mutant form of human galectin-9 having two carbohydrate recognition domains with a metal-binding site. Biochem Biophys Res Commun 490:1287–1293. doi:10.1016/j.bbrc.2017.07.009 28687490

[40] Zhang X, Song Z, Qin B, Zhang X, Chen L, Hu Y, Yuan Z. 2013. Rupintrivir is a promising candidate for treating severe cases of enterovirus-71 infection: evaluation of antiviral efficacy in a murine infection model. Antiviral Res 97:264–269. doi:10.1016/j.antiviral.2012.12.029 23295352

[41] Dong S, Shi Y, Dong X, Xiao X, Qi J, Ren L, Xiang Z, Zhou Z, Wang J, Lei X. 2022. Gasdermin E is required for induction of pyroptosis and severe disease during enterovirus 71 infection. J Biol Chem 298:101850. doi:10.1016/j.jbc.2022.101850 35339492 PMC9035723

[42] Golden-Mason L, McMahan RH, Strong M, Reisdorph R, Mahaffey S, Palmer BE, Cheng L, Kulesza C, Hirashima M, Niki T, Rosen HR. 2013. Galectin-9 functionally impairs natural killer cells in humans and mice. J Virol 87:4835–4845. doi:10.1128/JVI.01085-12 23408620 PMC3624298

[43] Iqbal AJ, Krautter F, Blacksell IA, Wright RD, Austin-Williams SN, Voisin MB, Hussain MT, Law HL, Niki T, Hirashima M, Bombardieri M, Pitzalis C, Tiwari A, Nash GB, Norling LV, Cooper D. 2022. Galectin-9 mediates neutrophil capture and adhesion in a CD44 and β2 integrin-dependent manner. FASEB J 36:e22065. doi:10.1096/fj.202100832R 34847625

[44] Onishi K, Fu HY, Sofue T, Tobiume A, Moritoki M, Saiga H, Ohmura-Hoshino M, Hoshino K, Minamino T. 2022. Galectin-9 deficiency exacerbates lipopolysaccharide-induced hypothermia and kidney injury. Clin Exp Nephrol 26:226–233. doi:10.1007/s10157-021-02152-2 34698914

[45] Wang Y, Dan K, Xue X, Yang X, Feng X, Yang Q, Yang J, Chen B. 2021. Translocating lipopolysaccharide correlates with the severity of enterovirus A71-induced HFMD by promoting pro-inflammation and viral IRES activity. Gut Pathog 13:69. doi:10.1186/s13099-021-00465-x 34809671 PMC8607650

[46] Machala EA, McSharry BP, Rouse BT, Abendroth A, Slobedman B. 2019. Gal power: the diverse roles of galectins in regulating viral infections. J Gen Virol 100:333–349. doi:10.1099/jgv.0.001208 30648945

[47] Vasta GR. 2009. Roles of galectins in infection. Nat Rev Microbiol 7:424–438. doi:10.1038/nrmicro2146 19444247 PMC3759161

[48] Levy Y, Auslender S, Eisenstein M, Vidavski RR, Ronen D, Bershadsky AD, Zick Y. 2006. It depends on the hinge: a structure-functional analysis of galectin-8, a tandem-repeat type lectin. Glycobiology 16:463–476. doi:10.1093/glycob/cwj097 16501058

[49] Zhou D, Zhao Y, Kotecha A, Fry EE, Kelly JT, Wang X, Rao Z, Rowlands DJ, Ren J, Stuart DI. 2019. Unexpected mode of engagement between enterovirus 71 and its receptor SCARB2. Nat Microbiol 4:414–419. doi:10.1038/s41564-018-0319-z 30531980

[50] Knowlton KU, Jeon ES, Berkley N, Wessely R, Huber S. 1996. A mutation in the puff region of VP2 attenuates the myocarditic phenotype of an infectious cDNA of the Woodruff variant of coxsackievirus B3. J Virol 70:7811–7818. doi:10.1128/JVI.70.11.7811-7818.1996 8892902 PMC190851

[51] Yeo H, Chong CWH, Chen EW, Lim ZQ, Ng QY, Yan B, Chu JJH, Chow VTK, Alonso S. 2022. A single amino acid substitution in structural protein VP2 abrogates the neurotropism of enterovirus A-71 in Mice. Front Microbiol 13:821976. doi:10.3389/fmicb.2022.821976 35369482 PMC8969769

[52] Wang H, Zhong M, Cui B, Yan H, Wu S, Wang K, Li Y. 2022. Neddylation of enterovirus 71 VP2 protein reduces its stability and restricts viral replication. J Virol 96:e0059822. doi:10.1128/jvi.00598-22 35510863 PMC9131864

[53] Phipps KM, Martinez A, Lu J, Heinz BA, Zhao G. 2004. Small interfering RNA molecules as potential anti-human rhinovirus agents: in vitro potency, specificity, and mechanism. Antiviral Res 61:49–55. doi:10.1016/j.antiviral.2003.08.005 14670593

[54] Wu Z, Yang F, Zhao R, Zhao L, Guo D, Jin Q. 2009. Identification of small interfering RNAs which inhibit the replication of several enterovirus 71 strains in China. J Virol Methods 159:233–238. doi:10.1016/j.jviromet.2009.04.002 19490979

[55] Li KL, Zhang XL, Dong DD, Zhu BN, Wang S, Wen XD, Cao WJ, Ru Y, Tian H, Zhu GL, He JJ, Guo JH, Dai JY, Zheng HX, Yang F, Zhu ZX. 2026. Picornavirus VP2 protein suppresses innate immunity through selective autophagic degradation of IKBKE/IKKε. Autophagy 22:330–350. doi:10.1080/15548627.2025.2597460 41319264 PMC12834145

[56] Samsa MM, Mondotte JA, Iglesias NG, Assunção-Miranda I, Barbosa-Lima G, Da Poian AT, Bozza PT, Gamarnik AV. 2009. Dengue virus capsid protein usurps lipid droplets for viral particle formation. PLoS Pathog 5:e1000632. doi:10.1371/journal.ppat.1000632 19851456 PMC2760139

[57] Belov GA, van Kuppeveld FJM. 2019. Lipid droplets grease enterovirus replication. Cell Host Microbe 26:149–151. doi:10.1016/j.chom.2019.07.017 31415743 PMC9004532

[58] Laufman O, Perrino J, Andino R. 2019. Viral generated inter-organelle contacts redirect lipid flux for genome replication. Cell 178:275–289. doi:10.1016/j.cell.2019.05.030 31204099 PMC7077330

[59] Melia CE, Peddie CJ, de Jong AWM, Snijder EJ, Collinson LM, Koster AJ, van der Schaar HM, van Kuppeveld FJM, Bárcena M. 2019. Origins of enterovirus replication organelles established by whole-cell electron microscopy. mBio 10:e00951-19. doi:10.1128/mBio.00951-19 31186324 PMC6561026

[60] Xiang Z, Tian Z, Wang G, Liu L, Li K, Wang W, Lei X, Ren L, Wang J. 2023. CD74 interacts with proteins of enterovirus D68 to inhibit virus replication. Microbiol Spectr 11:e0080123. doi:10.1128/spectrum.00801-23 37409968 PMC10434063

[61] Xiang Zichun, Gonzalez R, Wang Z, Ren L, Xiao Y, Li J, Li Y, Vernet G, Paranhos-Baccalà G, Jin Q, Wang J. 2012. Coxsackievirus A21, enterovirus 68, and acute respiratory tract infection, China. Emerg Infect Dis 18:821–824. doi:10.3201/eid1805.111376 22516379 PMC3358056

[62] Zhang YX, Huang YM, Li QJ, Li XY, Zhou YD, Guo F, Zhou JM, Cen S. 2017. A highly conserved amino acid in VP1 regulates maturation of enterovirus 71. PLoS Pathog 13:e1006625. doi:10.1371/journal.ppat.1006625 28938017 PMC5634653

[63] Dapat IC, Pascapurnama DN, Iwasaki H, Labayo HK, Chagan-Yasutan H, Egawa S, Hattori T. 2017. Secretion of galectin-9 as a DAMP during dengue virus infection in THP-1 cells. Int J Mol Sci 18:1644. doi:10.3390/ijms18081644 28788062 PMC5578034

